# The Selective Bromodomain and Extra‐Terminal Domain (BET) Inhibitor RVX‐208 Reduces Cocaine‐Seeking Behaviour and Alters Proteomic Pathways in the Nucleus Accumbens

**DOI:** 10.1111/adb.70121

**Published:** 2026-01-08

**Authors:** Tyler J. Sacko, Afshin Seyednejad, Jesse Engelhardt, Gregory C. Sartor

**Affiliations:** ^1^ Department of Pharmaceutical Sciences University of Connecticut Storrs Connecticut USA; ^2^ Center for Addiction Sciences and Innovation (CASI) University of Connecticut Storrs Connecticut USA; ^3^ Institute for the Brain and Cognitive Sciences (IBACS) University of Connecticut Storrs Connecticut USA

**Keywords:** BRD4, bromodomain and extra terminal domain, cocaine, dopaminergic signalling, proteomics, RVX‐208, self‐administration

## Abstract

Bromodomain and extra terminal domain (BET) epigenetic ‘reader’ proteins are key regulators of both behavioural and molecular responses to cocaine. In substance use disorder (SUD) models, BET function has primarily been investigated using small molecule inhibitors that prevent both bromodomains of BET proteins from interacting with acetylated histones. Although these inhibitors have been shown to be effective in SUD models, the potential adverse effects of pan‐BET inhibition may restrict translational applications. Recently, RVX‐208, a clinically tested and domain‐selective BET inhibitor, was found to reduce cocaine conditioned responses and cocaine‐induced gene expression in the nucleus accumbens (NAc), while avoiding the learning and memory impairments associated with pan‐BET inhibitors. However, the effectiveness of RVX‐208 in cocaine self‐administration procedures remains unclear. Here, we investigated whether repeated RVX‐208 treatment during abstinence altered cocaine‐seeking behaviour in rats trained to self‐administer cocaine. Male and female Sprague Dawley rats underwent 17 days of cocaine or sucrose self‐administration, followed by daily treatment with vehicle or RVX‐208 (25 mg/kg, ip) during a 14‐day abstinence period. Rats in the RVX‐208‐treated group showed reduced lever pressing compared to vehicle controls. Sucrose‐seeking and open field behaviour (distance travelled and time in the centre zone) were not significantly affected by RVX‐208 treatment. Proteomic analysis of the NAc revealed that RVX‐208 modulated several proteins, including those associated with dopamine activity (DRD1 and SLC6A3), transcriptional regulation (NFKB1), glutamate transport (SLC1A2) and ion channel activity (KCNJ10), and many changes were sex‐dependent. Collectively, these findings indicate that domain‐selective BET inhibition is effective at reducing cocaine‐seeking behaviour and point to novel mechanisms that may contribute to its therapeutic effect.

## Graphical abstract

Rats treated with the domain‐selective BET inhibitor RVX‐208 during a 2‐week forced abstinence period exhibited a reduction in cocaine‐seeking behaviour but not sucrose seeking or open field behaviour. This treatment effect was associated with proteomic changes in pathways relevant to dopaminergic and glutamatergic signalling in the nucleus accumbens. Together, these findings provide preliminary support for RVX‐208's therapeutic potential in cocaine self‐administration procedures and highlight potential mechanisms through which domain‐selective BET inhibition reduces cocaine‐seeking behaviour.

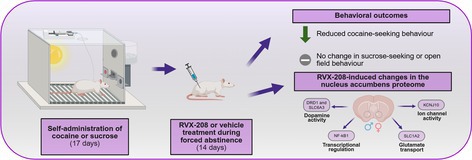

**Abbreviations**
BD1: bromodomain 1
BD2: bromodomain 1
BDNF: brain‐derived neurotrophic factor
BET: bromodomain and extra terminal domain
DRD1: dopamine receptor 1
FR1: fixed‐ratio 1
GO: Gene Ontology
ip: intraperitoneal
iv: intravenous
KEGG: Kyoto Encyclopedia of Genes and Genomes
LC/MS: liquid chromatography/mass spectrometry
NAc: nucleus accumbens
SUD: substance use disorder

## 1 Introduction

For multifactorial, polygenic disorders such as substance use disorders (SUDs), therapeutics targeting epigenetic mechanisms have emerged as a promising area of research [1]. Epigenetic drugs can modulate the proteins or complexes involved in writing, reading or erasing of DNA and histone modification, thereby influencing transcriptional activity. By restoring gene expression patterns disrupted by substance use, these therapies have the potential to mitigate the maladaptive neuroplasticity associated with drug craving and seeking behaviours. Indeed, several small molecule epigenetic inhibitors have been shown to reverse drug‐induced neuroadaptations and attenuate drug‐seeking behaviours in animal models of SUD [2, 3, 4]. Although these results are encouraging, the potential adverse effects of first‐generation, non‐selective epigenetic inhibitors may restrict their translational applications. Thus, for epigenetic pharmacotherapy to become a viable clinical option for SUD, further work is necessary to develop and test selective agents while also understanding the underlying mechanisms of the therapeutic response.

The development of potent, small molecule inhibitors targeting bromodomain and extra‐terminal (BET) proteins has generated substantial interest across a wide range of disease states [5, 6]. The BET family of epigenetic readers is comprised of four proteins (BRD2, BRD3, BRD4 and BRDT), each containing tandem bromodomains (BD1 and BD2) that recognize acetylated lysine residues on histone and non‐histone proteins [7]. When bound to histones, BET proteins serve as a key mediator between chromatin remolding and transcriptional activity [8]. First‐generation small molecule BET inhibitors, commonly referred to as pan‐BET inhibitors (e.g., JQ1, iBET‐151), exhibit high affinities for BD1 and BD2 across all BET protein members without binding to non‐BET bromodomains and have been shown to modulate disease‐associated gene expression in diverse cell types [9, 10]. The extensive preclinical efficacy of pan‐BET inhibitors in various disease contexts led to the rapid translation to clinical trials, particularly in oncology [11]. These discoveries have also spurred researchers to develop bromodomain‐selective BET therapeutics, several of which are now undergoing testing in a broad range of human diseases [12].

In preclinical SUD studies, BRD4 activity and expression are altered by cocaine exposure, and pan‐BET bromodomain inhibitors attenuate behavioural and transcriptional responses to cocaine [4, 13] and other misused substances [14, 15, 16]. However, the majority of studies on substance use disorders have utilized the pan‐BET inhibitor JQ1, a tool compound with poor pharmacokinetic properties and potential adverse effects that limit its suitability in clinical applications [17]. In the pursuit of more selective and safer therapeutic approaches for SUD, our lab recently found that systemic administration of RVX‐208 (also known as Apabetalone), a clinically tested, BD2‐selective BET inhibitor, attenuated cocaine conditioned place preference (CPP) without causing behavioural side effects associated with pan‐BET inhibitors [18]. Administration of RVX‐208 also reduced cocaine‐induced gene expression in the NAc without altering baseline expression, a mechanism distinct from pan‐BET inhibition [18]. However, most studies to date have focused on the effects of acute BET inhibitor administration on behaviour and gene expression, leaving the impact of repeated administration on behaviour and protein expression largely unexplored.

Building on these findings, the current studies aim to investigate whether repeated RVX‐208 treatment during abstinence affects cocaine‐seeking behaviour in self‐administration procedures, an approach with greater translational relevance to SUD. Additionally, proteomic analysis of the NAc was conducted to identify potential targets and pathways associated with RVX‐208 treatment. Together, these behavioural and molecular approaches provide the first assessment of RVX‐208's therapeutic potential in self‐administration procedures and shed light on potential mechanisms through which domain‐selective BET inhibition reduces cocaine‐seeking behaviour.

## 2 Materials and Methods

### 2.1 Drugs and Treatments

Cocaine HCl (National Institute on Drug Abuse Drug Supply Program) was dissolved in 0.9% sterile saline for self‐administration experiments. RVX‐208 (MedChemExpress) was dissolved in dimethyl sulfoxide (DMSO) and Tween 80 and then diluted with sterile saline to yield final concentrations of 10% DMSO and 5% Tween‐80 (v/v). RVX‐208 (25 mg/kg) or vehicle was administered by an intraperitoneal (ip) injection at a volume of 1 mL/kg. This dose was previously shown to be the lowest effective dose at reducing cocaine conditioned behaviour without affecting locomotor activity or learning and memory [18]. For jugular catheter surgeries, rats were anaesthetised with ketamine (80 mg/kg, ip) and xylazine (10 mg/kg, ip) mixture prior to surgery.

### 2.2 Animals

Male and female Sprague Dawley rats (8–10 weeks old; Charles River Laboratories; Wilmington, MA) were pair‐housed under a reverse 12 h/12 h light/dark cycle (lights off at 9 AM) and given ad libitum access to food and water. To determine appropriate sample sizes per experiment, power analysis was conducted based on effect sizes and variance estimates derived from previously published experiments. Rats were housed in a temperature‐controlled (22°C, 50% humidity) in an Association for Assessment and Accreditation of Laboratory Animal Care (AAALAC)‐accredited animal facility at the University of Connecticut and allowed to acclimate to the facility for at least 5 days before the experiment. All procedures were approved by the Institutional Animal Care and Use Committee of the University of Connecticut and performed in accordance with guidelines established by the US Public Health Service Policy on Humane Care and Use of Laboratory Animals.

### 2.3 Jugular Catheter Surgery

Rats were anaesthetised with a ketamine and xylazine mixture (80 and 10 mg/kg, ip). Once anaesthetised, an intravenous catheter made of sterile silastic tubing was implanted into the jugular vein and secured with sutures. The other end was routed subcutaneously over the shoulder to a cannula positioned on the back. Rats began self‐administration training after 5–7 days of recovery from surgery. Catheters were flushed after the self‐administration session with cefazolin (10 mg, iv) and heparin (10 U, iv).

### 2.4 Cocaine and Sucrose Self‐Administration

Rats were trained daily (2 h/day) to press the active lever in an operant chamber for intravenous delivery of cocaine (0.5 mg/kg/infusion, paired with light and tone cues) under fixed‐ratio 1 schedule of reinforcement (FR1 with 20‐s timeout after an infusion), as previously described [13]. For sucrose self‐administration studies, rats were trained to press the active lever for 45‐mg sucrose pellets (BioServ) under the same conditions as cocaine self‐administration. Inactive presses were recorded but had no consequences. All rats received 7 days of acquisition to meet the self‐administration criteria (at least two consecutive days with 12 infusions or pellets earned per day). For rats that did not press during the first three acquisition days, a wooden popsicle stick was affixed to the active lever to facilitate lever pressing. The sticks were removed prior to maintenance. Rats then received 10 maintenance days (2 h/day) of cocaine or sucrose self‐administration. After the last session, rats were returned to their home cage for 14 days of forced abstinence. During these 14 days, rats received a daily injection of vehicle or RVX‐208 (25 mg/kg, ip). This treatment regimen was chosen because BETs are known to regulate molecular factors (e.g., BDNF, glutamate receptors) [13, 19] that are known to be elevated during the first few weeks of abstinence and drive cocaine seeking behaviours [20, 21]. Additionally, early abstinence is a period when many individuals seek treatment and when relapse rates are elevated [22], making this treatment period relevant for translational studies. Twenty‐four hours after the last treatment, rats were returned to the operant chambers, and drug or sucrose seeking was measured during a 2‐h session. During this drug‐ or sucrose‐seeking test, lever presses were recorded, but an active lever press did not lead to drug or sucrose delivery or cues.

### 2.5 Open Field

The day after the last drug seeking test, open field behaviour was assessed in the same rats. The open field (Ugo Basile, product # 47150) consists of a square chamber with opaque grey walls. Distance travelled and time spent in the inner and outer zone were measured in a 30‐min test using EthoVision tracking software (Noldus).

### 2.6 Liquid Chromatography/Mass Spectrometry (LC/MS)

To assess sustained molecular changes following the treatments, tissue collection was performed 48 h after the last self‐administration test. Rats were briefly anaesthetised in a CO_2_ chamber prior to decapitation, and brains were collected over ice. The nucleus accumbens (NAc) was rapidly dissected and collected using a 2‐mm tissue punch and immediately frozen on dry ice. Tissue samples were submitted to the University of Connecticut Proteomics and Metabolomics Facility for untargeted protein identification and label‐free quantification by LC/MS. Sample lysates were quantified by BCA assay (Pierce part #23250), and 100 μg of each sample was aliquoted, adjusted to 2% SDS and processed with S‐Trap columns (ProtiFi LLC) using a modified commercial protocol. In brief, samples were reduced with dithiothreitol at 30°C for 1 h, alkylated with iodoacetamide for 30 min at 30°C in the dark, acidified with phosphoric acid and diluted by 6× with binding/wash buffer (90% methanol, 100‐mM TEAB). Samples were loaded onto S‐trap micro columns, washed with a total of 600‐μL binding/wash buffer and digested at 25°C overnight with trypsin/LysC at 1:10 enzyme:protein (w/w, Promega #V5073). Peptides were eluted with sequential washes of 50‐mM ammonium bicarbonate, 0.2% formic acid in water and 0.2% formic acid in 50% acetonitrile, dried to completion, desalted by reverse‐phase chromatography using Pierce peptide desalting spin columns (Thermo Fisher part #89852), dried to completion again and resuspended in Solvent A (0.1% formic acid in Fisher Optima LC/MS grade water). Final peptide concentration was quantified by A_280_ absorbance, and samples were normalized by total mass for injection.

Peptides were analysed in a randomized order using a Thermo Scientific Ultimate 3000 RSLCnano ultra‐high performance liquid chromatography (UPLC) system coupled to a Bruker timsTOF HT mass spectrometer. Each sample was injected onto a nanoEase M/Z Peptide BEH C18 column (1.7 μm, 75 μm × 250 mm, Waters Corporation) and separated by reversed‐phase UPLC using a gradient of 4%–30% Solvent B (0.1% formic acid in Fisher Optima LC/MS grade acetonitrile) over a 50‐min gradient at 300 nL/min flow, followed by a ramp of 30%–90% Solvent B over 10 min, for a 60‐min total gradient. Peptides were eluted directly into the HT using positive mode nanoflow electrospray ionization with a capillary voltage of 1500 V. Data were acquired using a modified data‐independent acquisition parallel accumulation‐serial fragmentation (DIA‐PaSEF) method with the following parameters: MS scans were taken over an *m*/*z* range of 100–1700, and a 1/K0 range of 0.7–1.4 V*s/cm^2^, with a rolling average of 10 scans. TIMS was enabled, with an intensity threshold of 5000, a ramp time of 100 ms and an accumulation time of 2 ms. MS2 scans were taken over a mass range of 400–1201 Da in 25‐Da windows with a 1‐Da overlap and over a mobility range of 0.6–1.43 1/K0. Cycle time was 1.8 s. Fragmentation energy was ramped from 20 to 59 eV over the mobility space.

Peptides and proteins were identified using Spectronaut (v19.9.250324, Biognosys) in directDIA mode, searching against the *Rattus Norvegicus* reference proteome (UP000002494, downloaded from UniProt on 2 January 2025) and the MaxQuant common contaminants database. Protease specificity was set to trypsin/P with a maximum of two missed cleavages, considering peptides from 7 to 52 residues in length. Up to five variable modifications per peptide were considered from the following list: protein N‐term acetylation, Asn/Gln deamidation, Gln conversion to pyro‐Glu and Met oxidation. Cys carbamidomethylation was set as a fixed modification. All FDR thresholds were 1% based on the target‐decoy method. Quantification was defined as area under the curve at the MS1 level, with no cross‐run normalization applied. All other parameters were left at default values. Common contaminant proteins like keratin and albumin identified from the common contaminants database were removed. Subsequent data analysis was done with msDiaLogue R package version 0.0.5 [23]. The mass spectrometry proteomics data have been deposited to the ProteomeXchange Consortium via the PRIDE [24] partner repository with the dataset identifier PXD068262.

### 2.7 Data Analysis

For behavioural studies, lever presses, distance travelled and time spent in centre were compared between groups using a two‐tailed Student's *t*‐test or analysis of variance (ANOVA). When a significant main effect was detected, Tukey's post hoc test was used for multiple comparisons. The percentage of body weight change following treatment was calculated as (Weight before treatment − Weight after treatment)/Weight before treatment × 100. Data are expressed as means ± SEM, and the level of significance was set at *p* < 0.05. Statistical analyses of behavioural studies were conducted using GraphPad Prism v8 (GraphPad Software, San Diego, California, USA).

For the LC/MS data analysis, abundance data were transformed to log_2_ values to better meet the assumptions of statistical models, such as equal variance and normally distributed residuals. Data were then normalized using Quantile normalization [25], and any remaining missing values imputed using the local minimum method (> 51% threshold) [26]. Next, differential protein abundance was analysed using two‐way ANOVA followed by Šidák correction to assess treatment, sex and treatment by sex differences. Proteins with a fold change ≥ 1.2 or ≤ 0.833 (|log2 fold change| ≥ 0.263) and *p*‐value < 0.05 (−log10 *p*‐values > 1.3) were considered statistically significant [27]. Volcano plots were generated using ggplot2 (v3.5.2) R package [28]. Upset plot was created via chiplot web application (chiplot.online/upset_plot.html). Enrichment analysis was performed using Enrichr web application (maayanlab.cloud/Enrichr) [29], and KEGG pathways or gene ontology (GO) molecular function terms with an adjusted *p*‐value (FDR) < 0.1 were considered significant, following the EnrichmentMap criteria for a moderately permissive threshold [30].

## 3 Results

### 3.1 The Effects of Repeated RVX‐208 Treatment on Cocaine‐ and Sucrose‐Seeking Behaviour

**FIGURE 1:**
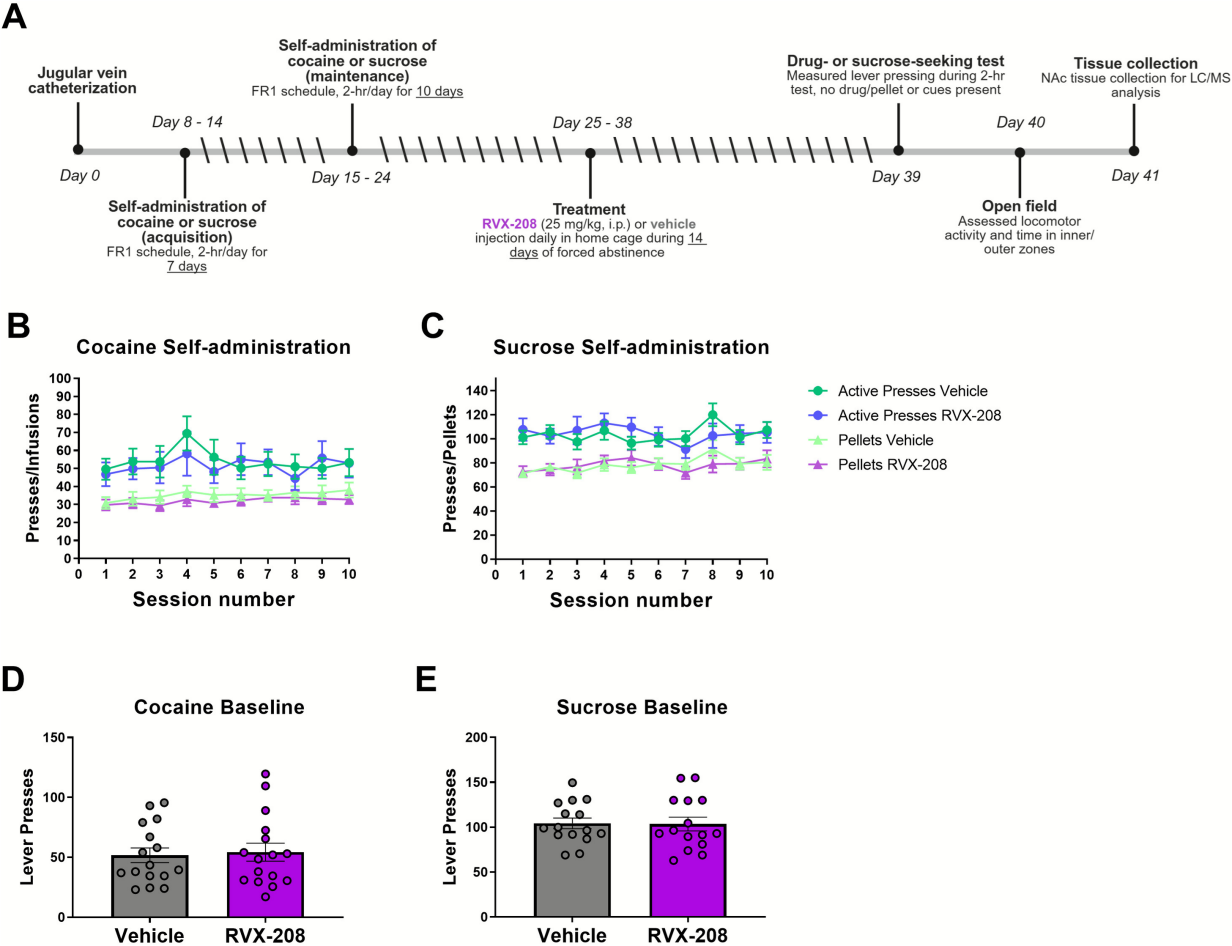
Experimental overview and cocaine and sucrose self‐administration data prior to treatment. (A) Experimental timeline. (B) Active lever presses and infusions during maintenance of cocaine self‐administration (vehicle, *n* = 16; RVX‐208, *n* = 16). (C) Active lever presses and pellets consumed during maintenance of sucrose self‐administration (vehicle, *n* = 15; RVX‐208, *n* = 15). (D) Prior to treatment, average active lever presses during the last 2 days of cocaine self‐administration. (E) Prior to treatment, average active lever presses during the last 2 days of sucrose self‐administration.

RVX‐208 was recently shown to reduce the acquisition of cocaine CPP and cocaine‐induced gene expression in the NAc [18]. Here, to build on these studies, we investigated the effects of repeated RVX‐208 treatment on cocaine‐ and sucrose‐seeking behaviour using self‐administration procedures. Male and female rats were trained to self‐administer cocaine or sucrose under an FR1 schedule for seven acquisition days and 10 maintenance days. Rats were then divided into treatment groups such that their average active lever presses, infusions and pellets consumed during the final 2 days of maintenance did not differ between groups (Figure 1). A two‐way ANOVA revealed sex differences in lever presses for cocaine but not sucrose during the last 2 days of maintenance (*F*_(1, 28)_ = 16.14, *p* = 0.0004) (Figure S1), but no group effect (*F*_(1, 28)_ = 0.1101, *p* = 0.7425) or group × sex interaction (*F*_(1, 28)_ = 0.1680, *p* = 0.6850). Rats then received daily intraperitoneal injections of RVX‐208 (25 mg/kg) or vehicle for 14 consecutive days in their home cage. Drug‐ and sucrose‐seeking behaviour were assessed by measuring lever presses during a drug‐ or sucrose‐free session without cues 24 h after the final day of treatment.

**FIGURE 2:**
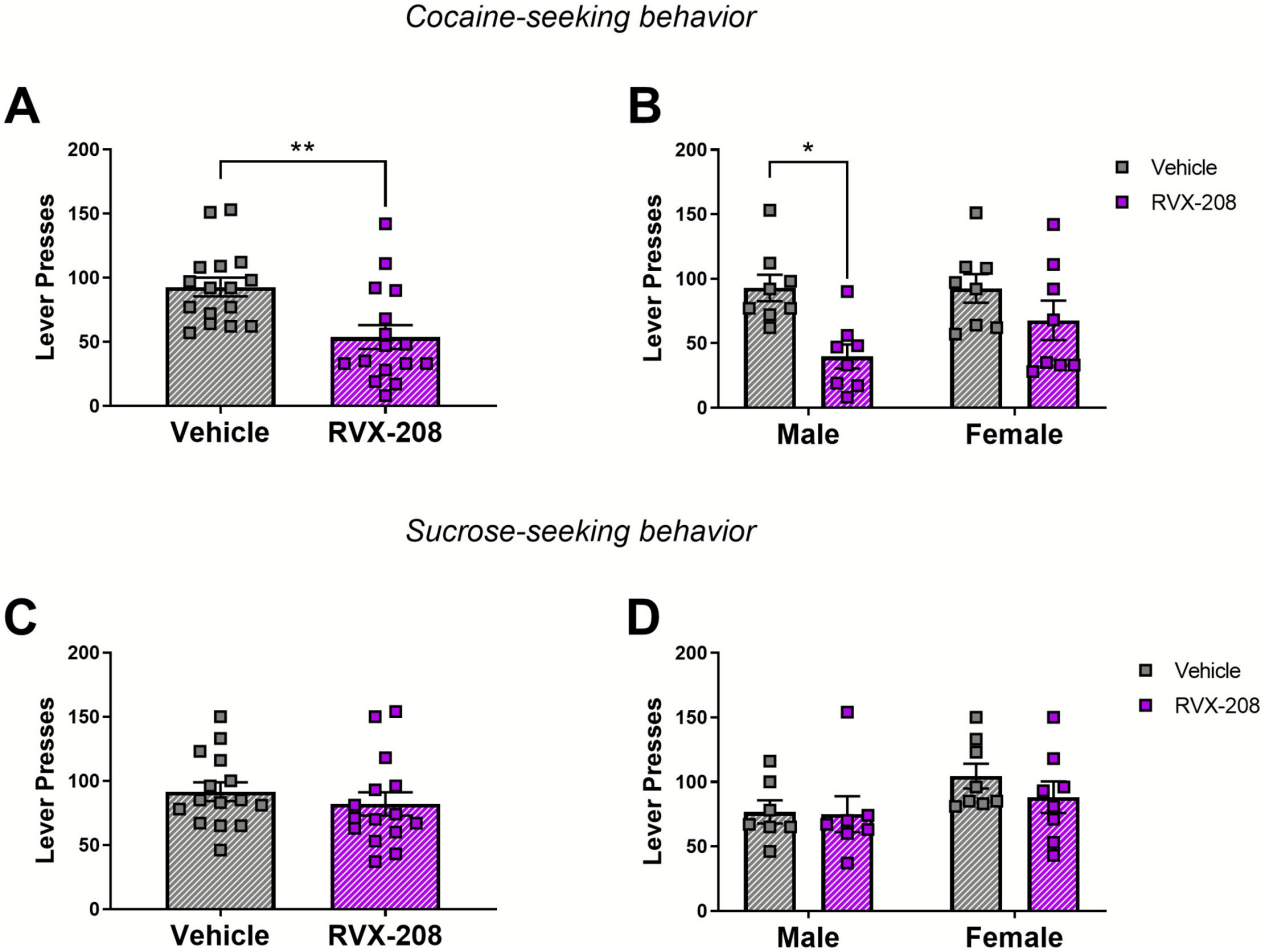
Repeated RVX‐208 treatment during abstinence reduces cocaine‐ but not sucrose‐seeking behaviour. (A) Active lever presses were measured during a drug‐free session without cues 24 h following the last vehicle (*n* = 16) or RVX‐208 (*n* = 16) treatment. (B) Post‐treatment lever presses separated by sex (*n* = 8m/8f per group). (C) Lever presses were measured during a sucrose‐free session without cues 24 h following the last vehicle (*n* = 15) or RVX‐208 (*n* = 15) treatment. (D) Post‐treatment lever presses separated by sex (*n* = 7m and 8f per group). Data are mean ± SEM. **p < 0.01 versus vehicle via Tukey's post hoc analysis.

In cocaine‐trained rats, RVX‐208 significantly reduced cocaine‐seeking behaviour compared to vehicle treatment (unpaired *t*‐test, *t*_(30)_ = 3.274, *p* = 0.0027) (Figure 2A). To examine sex‐dependent effects on treatment, a two‐way ANOVA revealed a significant effect of treatment (*F*_(1, 28)_ = 11.02, *p* = 0.0025), but no effect of sex (*F*_(1, 28)_ = 1.387, *p* = 0.2488) or treatment × sex interaction (*F*_(1, 28)_ = 1.463, *p* = 0.2365) (Figure 2B). Post hoc analysis indicated that RVX‐208 significantly attenuated lever pressing in male rats (*p* = 0.0168), but not female rats (*p* = 0.4554). To determine if RVX‐208 alters motivation for natural rewards, the same RVX‐208 or vehicle regimen was tested in sucrose self‐administration rats. In contrast to cocaine, RVX‐208 had no significant effect of treatment on sucrose‐seeking behaviour (unpaired *t*‐test, *t*_(28)_ = 0.8175, *p* = 0.4206) (Figure 2C), and two‐way ANOVA found no significant main effects for treatment (*F*_(1, 26)_ = 0.6343, *p* = 0.4330), sex (*F*_(1, 26)_ = 3.244, *p* = 0.0833), or treatment × sex interaction (*F*_(1, 26)_ = 0.4167, *p* = 0.5243) (Figure 2D). These results indicate that RVX‐208 reduces cocaine‐seeking behaviour without altering natural reward seeking behaviour.

### 3.2 The Effects of Repeated RVX‐208 Treatment on Open Field Behaviour and Weight

**FIGURE 3:**
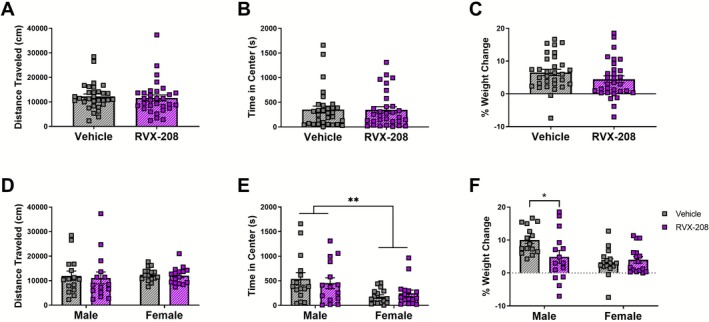
The effects of RVX‐208 on open field behaviours and body weight. (A) Locomotor activity (distance travelled) and (B) time spent in the centre zone of the open field were analysed in the same rats following repeated treatment with vehicle (*n* = 31) or RVX‐208 (*n* = 31). (C) Percentage change in weight following treatment with vehicle or RVX‐208. (D) Distanced travelled separated by sex (*n* = 15m and 16f per group). (E) Time in centre separated by sex. (F) Percentage of weight change separated by sex. Data are mean ± SEM. *p < 0.05 via Tukey's post hoc analysis, **p < 0.01 via two‐way ANOVA.

Open field behaviour (distance travelled and time spent in the centre) and changes in body weight were also measured in the same rats treated with vehicle or RVX‐208 (Figure 3). In the open field test, no significant group differences were found in distance travelled (unpaired *t*‐test, *t*_(60)_ = 0.3847, *p* = 0.7018) (Figure 3A) or time spent in centre (unpaired *t*‐test, *t*_(60)_ = 0.0755, *p* = 0.9401) (Figure 3B). Compared to vehicle, RVX‐208 did not alter body weight (unpaired *t*‐test, *t*_(60)_ = 1.458, *p* = 0.1500) (Figure 3C). To evaluate sex‐specific effects on treatment during open field, a two‐way ANOVA showed no significant main effect of treatment (*F*_(1, 58)_ = 0.1467, *p* = 0.7031), sex (*F*_(1, 58)_ = 0.1986, *p* = 0.6575) or treatment × sex interaction (*F*_(1, 58)_ = 0.01743, *p* = 0.8954) for total distance travelled (Figure 3D). For time spent in the centre, a significant effect was observed for sex (*F*_(1, 58)_ = 9.919, *p* = 0.0026) but not treatment (*F*_(1, 58)_ = 0.01168, *p* = 0.9143) or treatment × sex interaction (*F*_(1, 58)_ = 0.7197, *p* = 0.3997) (Figure 3E). For body weight, a two‐way ANOVA revealed a significant main effect of sex (*F*_(1, 58)_ = 8.610, *p* = 0.0048) and a significant treatment × sex interaction (*F*_(1, 58)_ = 5.023, *p* = 0.0289), but no main effect of treatment (*F*_(1, 58)_ = 2.771, *p* = 0.1014) (Figure 3F). In post hoc analysis, the percentage of weight change was reduced by RVX‐208 treatment compared to vehicle in males (*p* = 0.0418), but no significant change was observed in females.

### 3.3 Proteomic Analysis of the Nucleus Accumbens Following Treatment With RVX‐208

**FIGURE 4:**
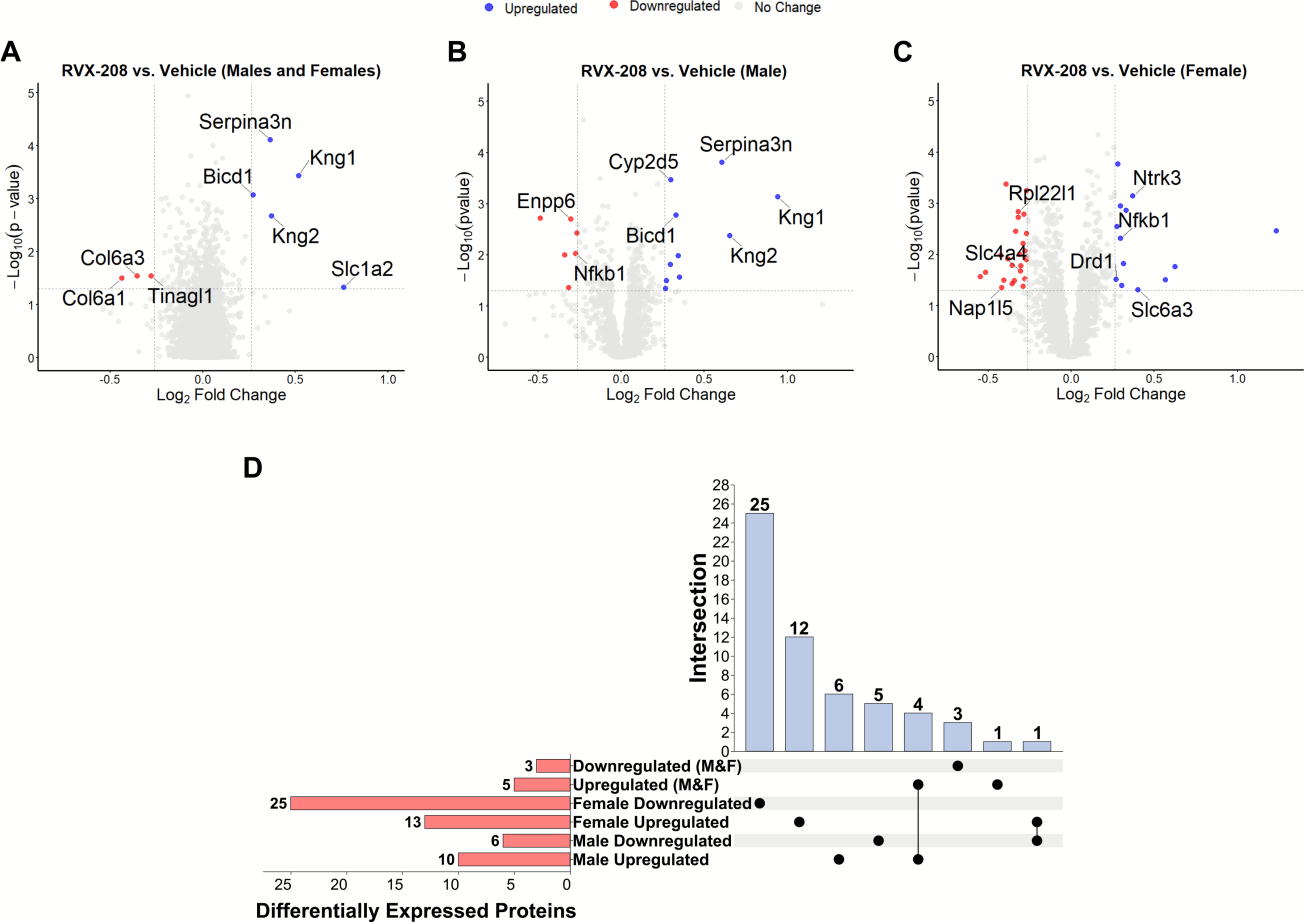
Differential protein expression in the NAc following RVX‐208 treatment. Volcano plots showing differentially expressed proteins between RVX‐208 and vehicle quantified via LC–MS/MS in (A) males and females combined, (B) males only and (C) females only. Significantly upregulated proteins are shown in blue, downregulated in red and unchanged in grey. (D) Upset plot showing overlap between differentially expressed proteins in males and females, including proteins uniquely or commonly upregulated or downregulated. Bars indicate the number of proteins shared between groups, while dots indicate the specific sets intersecting. Vehicle: *n* = 16 (male *n* = 8, female *n* = 8); RVX‐208: *n* = 16 (male *n* = 8, female *n* = 8).

To assess RVX‐208‐induced changes in protein expression, mass spectrometry‐based untargeted protein identification and label‐free quantification was performed on the NAc tissue from cocaine self‐administration rats treated with RVX‐208 or vehicle. A complete list of analysed proteins is provided in File S1. Differentially expressed proteins were visualized using volcano plots of the log2 fold‐change (x‐axis) versus −log10 *p*‐value (y‐axis) shown for all animals combined (Figure 4A), males only (Figure 4B) and females only (Figure 4C). In the combined analysis of males and females, RVX‐208 treatment significantly increased the abundance of 5 proteins and decreased three proteins relative to vehicle. When analysed by sex, RVX‐208 treatment in males significantly upregulated 10 proteins and downregulated six proteins, whereas in females, RVX‐208 significantly upregulated 13 proteins and downregulated 25 proteins compared with vehicle. The differentially expressed proteins included those involved in dopamine signalling (DRD1 and SLC6A3), transcriptional regulation (NFKB1), glutamate transport (SLC1A2) and ion channel activity (KCNJ10) [31]. An upset plot (Figure 4D) demonstrated largely distinct sets of differentially expressed proteins in males and females, with minimal overlap. KEGG pathway and GO molecular function analyses (Table 1) of the differentially expressed proteins indicated enrichment in terms such as Cocaine addiction, Amphetamine addiction, Neurotrophin Binding, Monoamine Transmembrane Transporter Activity and Transcription Coactivator Binding, among others (see Files S2A and S2B for the top 10 significant terms in males and females).

**TABLE 1:** Select KEGG pathway analysis and gene ontology (GO) annotations that were enriched in the NAc of male or female rats.

Name	*p*	Adjusted *p*	Odds ratio	Combined score	Group and direction of change	Database
Inositol phosphate metabolism	0.03591	0.08309	30.74	102.25	Male‐upregulation	KEGG
Inositol hexakisphosphate kinase activity (GO:0000828)	0.003495	0.02908	370.07	2093.27	Male‐upregulation	GO Molecular Function
Inositol phosphate kinase activity (GO:0180030)	0.003994	0.02908	317.19	1751.86	Male‐upregulation	GO Molecular Function
Arachidonate epoxygenase activity (GO:0008392)	0.006482	0.02908	184.98	932.06	Male‐upregulation	GO Molecular Function
Cocaine addiction	0.01461	0.0731	83.11	351.21	Male‐downregulation	KEGG
Transcription coactivator binding (GO:0001223)	0.01075	0.04392	114.05	516.95	Male‐downregulation	GO Molecular Function
Cocaine addiction	3.885E−06	0.0003846	130.05	1620.23	Female‐upregulation	KEGG
Calcium signalling pathway	0.0004465	0.01641	25	192.85	Female‐upregulation	KEGG
Amphetamine addiction	0.0008928	0.01819	54.06	379.54	Female‐upregulation	KEGG
Neurotrophin binding (GO:0043121)	0.004542	0.03776	277.51	1497.03	Female‐upregulation	GO Molecular Function
Ribonuclease P RNA binding (GO:0033204)	0.005836	0.03776	208.11	1070.48	Female‐upregulation	GO Molecular Function
Ribonuclease P activity (GO:0004526)	0.006482	0.03776	184.98	932.06	Female‐upregulation	GO Molecular Function
Monoamine transmembrane transporter activity (GO:0008504)	0.007129	0.03776	166.47	822.99	Female‐upregulation	GO Molecular Function

## 4 Discussion

Novel therapeutics for SUDs are urgently needed given the scarcity of effective medications and the high rates of relapse. RVX‐208 is a promising drug candidate for translational SUD research because it has been clinically tested in other disease states and displays a more favourable safety profile compared to other pan‐BET inhibitors that have been tested in SUD models [32, 33]. Here, we revealed that repeated administration of RVX‐208 during a 2‐week forced abstinence period attenuates cocaine seeking in a self‐administration procedure. In parallel, we identified RVX‐208‐mediated proteomic changes in the NAc. These findings highlight the potential long‐term molecular impact of domain‐selective BET inhibitor treatment and build on previous data showing that RVX‐208 reduces acquisition of cocaine CPP and cocaine‐induced gene expression in the NAc [18].

Previous studies using the pan‐BET inhibitor JQ1 reported a reduction in heroin self‐administration and cue‐induced reinstatement following intra‐striatal treatment [14], as well as decreased cocaine‐induced reinstatement after intra‐accumbal injections [4]. Unlike prior SUD studies, which relied on intracranial administration of a pan‐BET inhibitor during maintenance or before reinstatement, the current study demonstrates that repeated systemic administration of RVX‐208 during forced abstinence effectively reduces cocaine seeking without altering sucrose seeking or open field behaviour. This indicates that RVX‐208's effects are not broadly disrupting natural reward processing or locomotor activity. Although RVX‐208 reduced cocaine seeking when males and females were analysed together, only males showed a significant effect when examined separately. Proteomic results also showed sex‐dependent effects, revealing largely distinct sets of differentially expressed proteins in males versus females, with minimal overlap and even opposing regulation of certain proteins. In previous studies, RVX‐208 reduced acquisition of cocaine CPP to a similar degree in male and female mice, yet its impact on mRNA expression of specific cocaine‐induced genes differed between sexes [18]. Thus, our current results align with a growing body of literature demonstrating sex differences in epigenetic regulation and drug‐seeking behaviours [34, 35, 36]. While the current study was not intended to fully characterize sex‐dependent effects, future research is needed to evaluate whether adjustments in dosing, timing or other strategies may enhance the efficacy in both female and male rats.

In other results, male, but not female, rats treated with RVX‐208 gained less weight compared to vehicle‐treated controls. Similar to the current results, a previous study showed that male mice treated with RVX‐208 and fed a high‐fat diet did not gain as much weight as vehicle‐treated mice despite no decrease in food intake [37]. These findings combined with the current data suggest that RVX‐208 may reduce weight gain by altering peripheral metabolic pathways rather than by suppressing appetite. Further supporting this idea, the pan‐BET inhibitor JQ1 was found to reduce fat mass and diet‐induced weight gain without altering skeletal muscle mass and strength [38]. In clinical studies with RVX‐208, no change in body mass index was reported [39], and the only notable safety concern was reversible liver‐enzyme elevations occurring in approximately 7% of patients [40]. Overall, while the reduced weight gain in males was modest, future studies should investigate the extent to which RVX‐208 alters peripheral metabolism and whether such effects contribute to or interact with the therapeutic efficacy of RVX‐208 in cocaine use disorder.

Previous studies have shown that BET proteins regulate transcription induced by dopamine receptor 1 (DRD1) stimulation, and BET inhibition or *Brd4* knockdown blocks DRD1‐induced transcription in primary striatal neurons [41]. Notably, we observed an upregulation of DRD1 and sodium‐dependent dopamine transporter (SLC6A3, also known as DAT1) proteins in female rats but not in male rats treated with RVX‐208. This sex‐specific upregulation could underlie distinct behavioural or pharmacological responses to RVX‐208. It is well established that there is a significant sex dichotomy in the neurobiological and behavioural effects of cocaine, with females showing greater vulnerability both in human and animal studies [42]. Consequently, elevations of DRD1 and DAT in female, but not male, rats treated with RVX‐208 may explain the weaker therapeutic response in female rats, as DRD1 and DAT upregulation has been previously associated with increased cocaine seeking behaviours [43, 44]. RVX‐208 treatment also reduced NFKB1 in males and increased NFKB1 in females relative to vehicle. Interestingly, BRD4 is known to interact with RelA, a subunit of NFKB1 [10], and previous studies have shown that repeated cocaine exposure increases NFKB1 and its target genes while *Nfkb1* knockdown prevents the rewarding effects of cocaine [45]. This pattern may account for the sex‐specific differences we observed in NFKB1 expression and could help explain the divergent behavioural outcomes in self‐administration studies, where RVX‐208 reduced cocaine seeking in males but had no effect in females.

It is known that cocaine induces widespread adaptations in glutamatergic signalling within the NAc that underlies relapse [46]. Repeated cocaine use disrupts the balance between synaptic and extrasynaptic glutamate levels, leading to elevated glutamatergic transmission that drives drug‐seeking behaviours [47]. A major contributor to this imbalance is the downregulation of the astrocytic glutamate transporter SLC1A2 (GLT‐1), which accounts for 90% of synaptic glutamate clearance [48]. Reduced SLC1A2 expression has been consistently reported following cocaine self‐administration [49, 50], and pharmacological or genetic restoration of SLC1A2 function reliably decreases reinstatement of cocaine seeking [49, 51, 52]. Notably, our proteomics analysis revealed upregulation of SLC1A2 expression in RVX‐208 treated rats, which parallels the overall reduction in cocaine seeking observed in our behavioural experiments. Together, these findings suggest that BET‐mediated regulation of proteins involved in glutamatergic homeostasis may represent another key mechanism through which BET inhibition reduces cocaine‐seeking behaviour.

In summary, the present study demonstrates that repeated systemic administration of RVX‐208 during forced abstinence reduces cocaine‐seeking without altering sucrose seeking and open field behaviour. Additionally, the treatment effect was associated with proteomic changes in pathways relevant to dopaminergic and glutamatergic signalling in the NAc. These findings extend on prior evidence supporting domain‐selective BET inhibition as a promising strategy to reduce cocaine conditioned behaviours, while highlighting sex‐dependent effects that warrant further investigation. Limitations of the study include lack of sub‐region (shell vs. core) and cell type‐specific analysis of the NAc, the absence of functional mechanistic studies, and the evaluation of a single dose and treatment timepoint. Although seeking behaviour was not directly compared between cocaine and sucrose groups, the absence of a sham catheter surgery in the sucrose group represents a procedural difference that may be viewed as a limitation. Taken together, these results provide preliminary support for the efficacy of RVX‐208 in cocaine self‐administration procedures, though additional testing is needed to fully establish its potential as a therapeutic for cocaine use disorder.

## Author Contributions

A.S., T.J.S. and G.C.S. designed the research, and A.S., T.J.S. and J.E. performed the experiments and analysed the data. A.S., T.J.S. and G.C.S. interpreted the data. T.J.S. and A.S. prepared the figures. T.J.S., A.S. and G.C.S. wrote the manuscript.

## Funding

Research reported in this publication was supported by the National Institute on Drug Abuse of the National Institutes of Health under Award Number R01DA058700 and the Connecticut Institute for the Brain and Cognitive Sciences (IBACS).

## Ethics Statement

Animal experiments were approved by the University of Connecticut Institutional Animal Care and Use Committee (22‐027).

## Consent

Authors have reviewed the manuscript and consent to publish.

## Conflicts of Interest

The authors declare no conflicts of interest.

## Supporting information


**Figure S1:** Cocaine and sucrose self‐administration data prior to treatment, separated by sex. (A) Male active lever presses and infusions during maintenance of cocaine self‐administration (vehicle, *n* = 8; RVX‐208, *n* = 8). (B) Female active lever presses and infusions during maintenance of cocaine self‐administration (vehicle, *n* = 8; RVX‐208, *n* = 8). (C) Male active lever presses and pellets consumed during maintenance of sucrose self‐administration (vehicle, *n* = 7; RVX‐208, *n* = 7). (D) Female active lever presses and pellets consumed during maintenance of sucrose self‐administration (vehicle, *n* = 8; RVX‐208, *n* = 8). Average active lever presses during the last 2 days of cocaine (E) and sucrose (F) self‐administration, separated by sex. Data are mean ± SEM. ***p < 0.001 indicates sex differences via two‐way ANOVA.

**File S1:** Mass spectrometry‐quantified protein abundance analysis in the NAc of male and female rats treated with RVX‐208 versus vehicle. The table lists all proteins quantified by mass spectrometry in the nucleus accumbens. Columns include estimated marginal means for male RVX‐208 versus control and female RVX‐208 versus control, family‐wise error–adjusted *p*‐values for males and females, group means for RVX‐208 and control and *p*‐values for sex, treatment and sex × treatment interaction.

**File S2:** Top enriched KEGG pathways and GO molecular function terms in the NAc of male and female rats. The table reports the top 10 KEGG pathways and top 10 Gene Ontology (GO) molecular function terms identified for (A) males and (B) females following RVX‐208 or vehicle treatment. Pathways and terms are ranked by significance (adjusted *p*‐value or FDR), with associated enrichment statistics provided. These data complement Table 1, which lists select pathways and GO annotations.

## Data Availability

Data related to this project may be available upon a reasonable request. Mass spectrometry data have been deposited to the ProteomeXchange Consortium via the PRIDE with the dataset identifier PXD068262.
