# Supplementary material for: The Selective Bromodomain and Extra‐Terminal Domain (BET) Inhibitor RVX‐208 Reduces Cocaine‐Seeking Behaviour and Alters Proteomic Pathways in the Nucleus Accumbens
Source: Addict Biol. 2026 Jan 8;31(1):e70121. doi: 10.1111/adb.70121 (PMC12783071; doi:10.1111/adb.70121)
Supplement: Supplementary file 1 — Figure S1: Cocaine and sucrose self‐administration data prior to treatment, separated by sex. (A) Male active lever presses and infusions during maintenance of cocaine self‐administration (vehicle, n = 8; RVX‐208, n = 8). (B) Female active lever presses and infusions during maintenance of cocaine self‐administration (vehicle, n = 8; RVX‐208, n = 8). (C) Male active lever presses and pellets consumed during maintenance of sucrose self‐administration (vehicle, n = 7; RVX‐208, n = 7). (D) Female active lever presses and pellets consumed during maintenance of sucrose self‐administration (vehicle, n = 8; RVX‐208, n = 8). Average active lever presses during the last 2 days of cocaine (E) and sucrose (F) self‐administration, separated by sex. Data are mean ± SEM. ***p < 0.001 indicates sex differences via two‐way ANOVA. [file ADB-31-e70121-s002.docx]

**The selective bromodomain and extra-terminal domain (BET) inhibitor RVX-208 reduces cocaine-seeking behavior and alters proteomic pathways in the nucleus accumbens**

**Tyler J. Sacko****^1,2,3^, Afshin Seyednejad^1,2,3^, Jesse Engelhardt^1^, Gregory C. Sartor^1,2,3^***

1. Department of Pharmaceutical Sciences, University of Connecticut, Storrs, CT 06269, USA

2. Center for Addiction Science and Innovation (CASI), University of Connecticut, Storrs, CT 06269, USA

3. Institute for the Brain and Cognitive Sciences (IBACS), University of Connecticut, Storrs, CT 06269, USA

*Correspondence to:

Gregory C. Sartor, Ph.D.

Department of Pharmaceutical Sciences

University of Connecticut

69 N. Eagleville road, Storrs, CT 06269

e-mail: Gregory.sartor@uconn.edu

Telephone: 860-486-3655

**Supplementary Legends**

**Supplementary Figure 1. Cocaine and sucrose self-administration data prior to treatment, separated by sex. (A)** Male active lever presses and infusions during maintenance of cocaine self-administration (vehicle, n = 8; RVX-208, n = 8). (**B**) Female active lever presses and infusions during maintenance of cocaine self-administration (vehicle, n = 8; RVX-208, n = 8). (**C**) Male active lever presses and pellets consumed during maintenance of sucrose self-administration (vehicle, n = 7; RVX-208, n = 7). (**D**) Female active lever presses and pellets consumed during maintenance of sucrose self-administration (vehicle, n = 8; RVX-208, n = 8). Average active lever presses during the last two days of cocaine (**E**) and sucrose (**F**) self-administration, separated by sex. Data are mean ± SEM. ***P < 0.001 indicates sex differences via two-way ANOVA.

**Supplementary File 1. Mass spectrometry-quantified protein abundance analysis in the NAc of male and female rats treated with RVX-208 vs. Vehicle.** The table lists all proteins quantified by mass spectrometry in the nucleus accumbens. Columns include estimated marginal means for Male RVX-208 vs. control and Female RVX-208 vs. control, family-wise error–adjusted p-values for males and females, group means for RVX-208 and control, and p-values for sex, treatment, and sex × treatment interaction.

**Supplementary File 2. Top enriched KEGG pathways and GO molecular function terms in the NAc of male and female rats.** The table reports the top 10 KEGG pathways and top 10 Gene Ontology (GO) molecular function terms identified for (A) males and (B) females following RVX-208 or vehicle treatment. Pathways and terms are ranked by significance (adjusted p-value or FDR), with associated enrichment statistics provided. These data complement Table 1, which lists select pathways and GO annotations.

**Supplementary Figure 1**

**
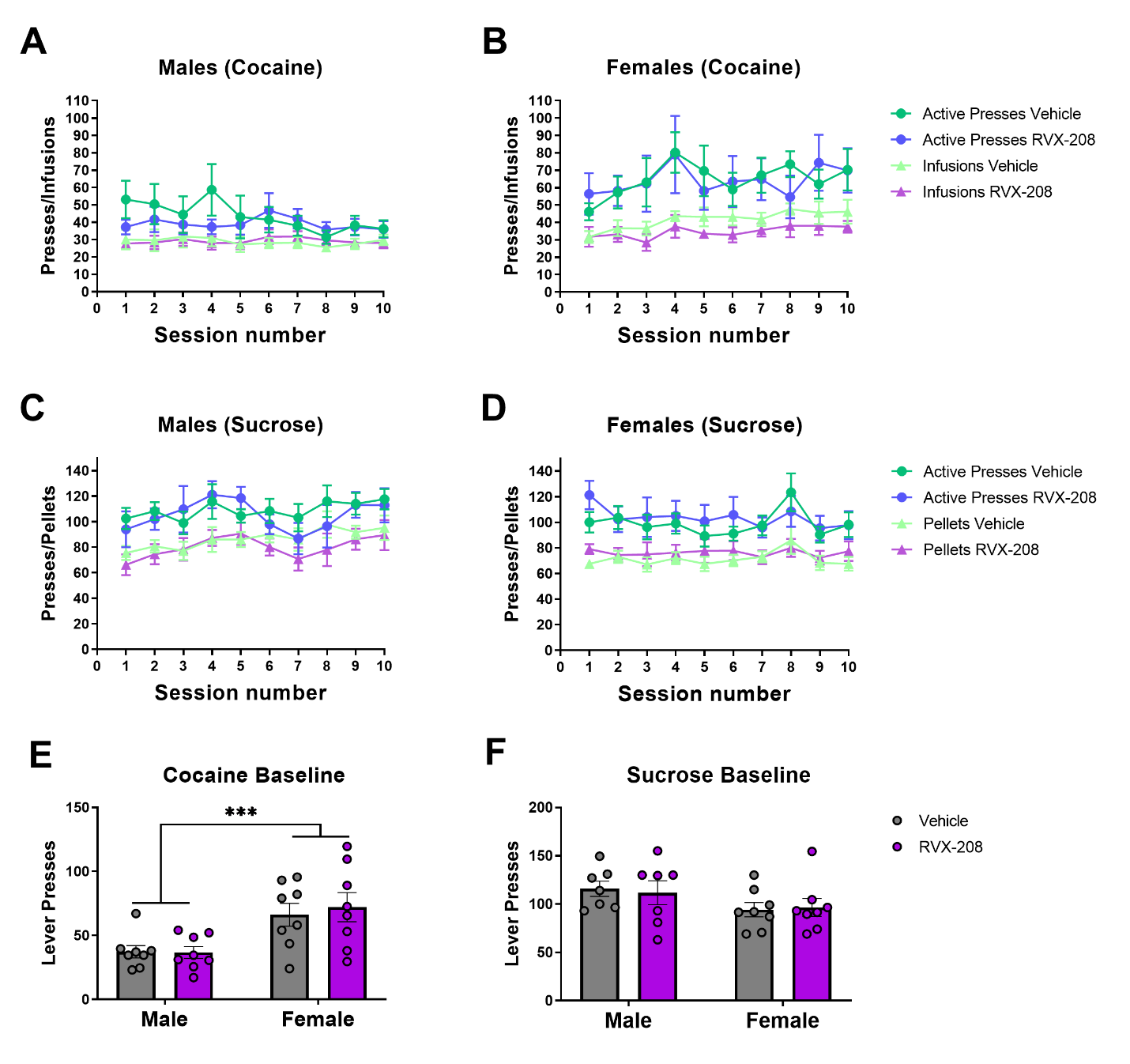
**
